# Increased lumbar puncture success using a paramedian approach: A retrospective cohort study

**DOI:** 10.1002/jhm.70138

**Published:** 2025-07-21

**Authors:** Jacob Wagner, Kiril Dimitrov, Zachary Lauer, Swati Jain, Omar Jibril, Zach Kaltenborn, Aditya Kler, Andrew Olson, Justin Clark, Kyle Rudser, Matthew Yocum

**Affiliations:** ^1^ Division of Hospital Medicine University of Minnesota Minneapolis Minnesota USA; ^2^ Division of Biostatistics & Health Data Science University of Minnesota Minneapolis Minnesota USA; ^3^ Division of Hospital Medicine University of California San Francisco San Francisco California USA

## Abstract

**Background:**

Lumbar puncture (LP) is frequently necessary in hospitalized patients for diagnostic and therapeutic purposes. Traditional landmark identification by palpation of the lumbar spine and pelvis can see significant failure rates. A paramedian approach is an accepted, though less frequently used technique associated with higher success rates for spinal access in anesthesia when aided by ultrasound. Similarly, point‐of‐care ultrasound (POCUS) of the paramedian acoustic window may help guide a paramedian LP technique.

**Objective:**

To compare the probability of success before and after the implementation of a standardized training curriculum for lumbar punctures (LPs) performed by the Hospital Medicine Procedure Service (HMPS).

**Methods:**

The HMPS at the University of Minnesota Medical Center (UMMC), a large tertiary academic medical center, implemented a standardized training for an ultrasound‐assisted paramedian (USPM) approach to all bedside lumbar punctures (LPs) and then conducted a retrospective review comparing success rates between the traditional midline (ML) and USPM approaches.

**Results:**

A total of 269 LPs were performed on hospitalized patients. Before standardization, the probability of a successful LP was 72.6% using ML approach. After standardization, using a USPM approach, the probability of a successful LP was 85.4%. The probability of a successful procedure was 13.6 (95% confidence interval: 2.8, 24.4, *p* = .014) percentage points higher for the USPM approach when compared to the ML approach and accounting for sex, age, BMI, and if the LP was for chemotherapy.

**Conclusions:**

Institutions with a HMPS should consider transitioning to use of the USPM approach for bedside LPs, which is associated with a higher probability of success and reduced utilization of more resource‐intensive hospital services, such as Interventional Radiology or Neuroradiology.

## INTRODUCTION

Lumbar punctures (LPs) are commonly needed among hospitalized patients. Timely and successful LPs are necessary in the evaluation of many conditions, such as central nervous system infections, encephalopathy, and malignancy. Hospitalists and emergency department clinicians are frequently tasked with performing LPs. Traditionally, most clinicians learn to perform this bedside procedure using a landmark‐guided, midline (ML) approach which involves palpation of the lumbar spine. Notably, the ML approach is associated with clinically meaningful failure rates, generally reported at 20%–30%.^1^

While the use of point of care ultrasound (POCUS) assistance for a ML approach is associated with increased LP success rates, primarily in patients with a high body mass index (BMI) and without palpable landmarks, unsuccessful attempts are still common. Alternative LP techniques may offer additional advantages. Off‐midline or paramedian approaches to LPs have been used before the incorporation of ultrasound (US) and the use of paramedian ultrasound has been shown to improve spinal anesthesia success efficiency using both ultrasound‐guided (active visualization of needle using POCUS during needle insertion) and ultrasound‐assisted (static assessment using POCUS followed by a “blind” needle insertion without the use of POCUS) techniques.^2^, ^3^ The needle needs to be directed at the correct sagittal‐oblique angle, which can vary considerably between patients and can be difficult to determine without the aid of US. The paramedian approach using ultrasound likely adds value as seen in the anesthesia literature by identifying the angle needed to access the subarachnoid space and the optimal spinal level (most open spinal level without bony obstruction). However, evidence that an ultrasound‐assisted paramedian approach (USPM) utilizing these views can improve the success rate for bedside LPs is lacking.

Many hospitalists that perform LPs at the bedside are familiar with POCUS use, and have learned US‐assisted techniques for commonly performed procedures such as paracentesis, making investigation of new US‐assisted techniques for bedside procedures increasingly relevant. This is especially true for centers with a hospital medicine procedure service (HMPS); while the general prevalence of US use for LPs is unknown and its recommendation as the standard of care is debated,^4^ a HMPS at a single center recently demonstrated a 76.7% US use rate.^5^ Developing techniques that improve success rates is increasingly important, since failed LP attempts can result in delays in care and add burden to neuroradiology services that are needed to perform other vital procedures that cannot be performed at the bedside. In this context, and following the Society of Hospital Medicine position statement recommending US‐assistance for LPs,^6^ the HMPS at the University of Minnesota Medical Center (UMMC) adopted an USPM LP approach. This study aims to assess the impact of standardizing an USPM approach to performing bedside LPs in comparison to a ML approach for a HMPS in an acute care setting.

## METHODS

### Setting

UMMC is a large, urban, tertiary academic medical center with over 300 beds. Since 2016, UMMC has had an active HMPS that consists of 1 attending hospitalist and 0–1 trainees who are available 8:00 a.m. to 5:00 p.m., 7 days a week. A substantial portion of the LPs in the hospital are performed by the HMPS, and while other services (Neurology, Emergency Medicine, Critical Care, Hospital Medicine, and Oncology) may perform bedside LPs, this occurs infrequently. At inception, use of US during LPs was uncommon and most LPs were performed using the traditional landmark‐based ML approach. Unsuccessful bedside LPs are often referred to neuroradiology for fluoroscopic or CT guidance.

### Study design

**Figure 1:**
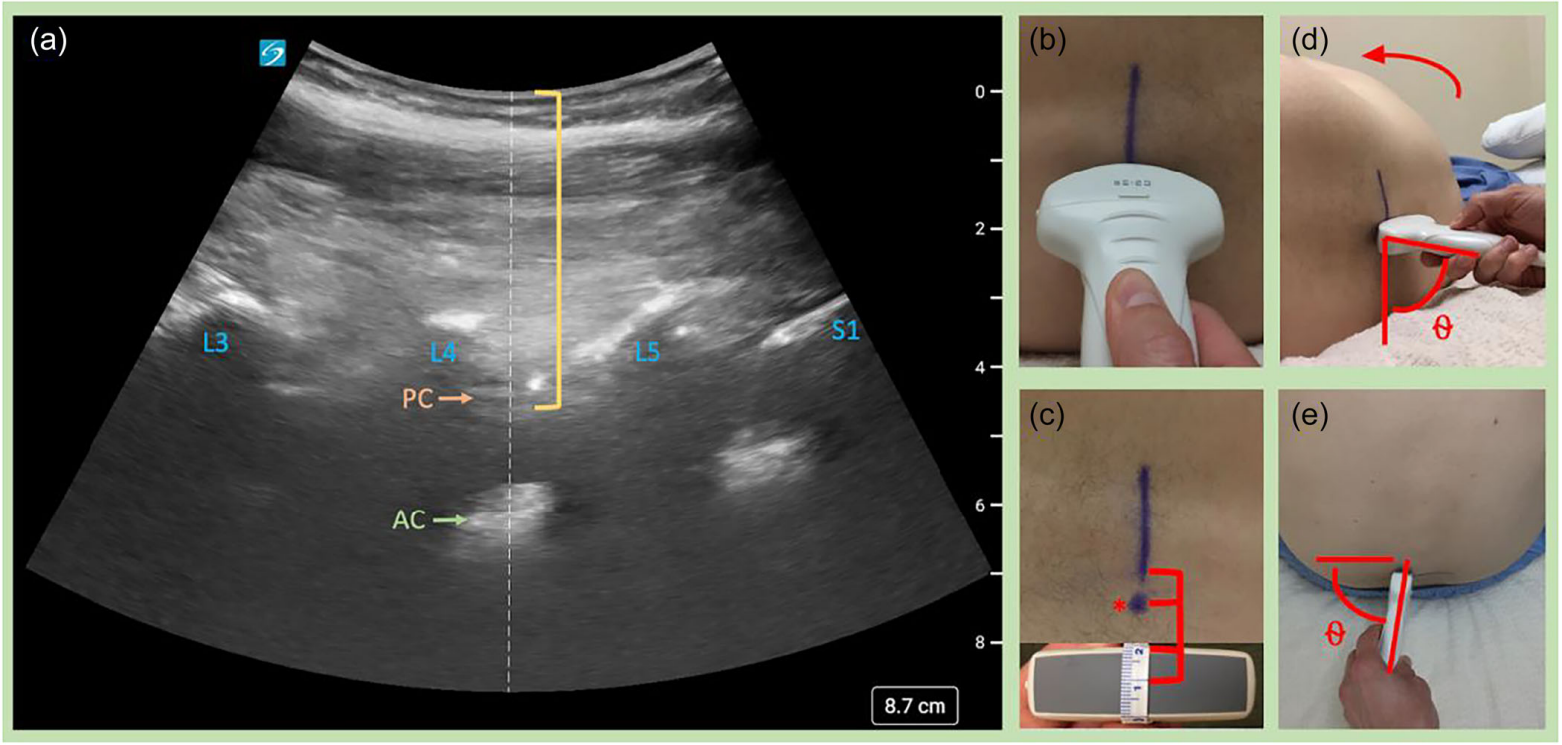
Paramedian‐assisted ultrasound diagram. (a) Ultrasound image of the paramedian structures at L4/5 (patient's left in this case), demonstrating the lamina (L3–S1), and spinal canal structures (PC‐posterior complex: ligamentum flavum, epidural space and posterior dura, and AC‐anterior complex: anterior dura and posterior longitudinal ligament), with estimated needle length just beyond the PC represented by the brackets. Needle entry is centered on the anterior complex. (b, c) Mark the spinal space along the center of the probe centered over the AC as demonstrated in (b) and then a mark (dot) is made at the anticipated center point of the transducer which indicates the needle entry. This is typically ~1 cm from the bottom of the line and from the patient's midline. (d, e) Demonstrates the angle of entry, which should be nearly orthogonal (~75–80°) to the coronal plane. (d) It is important to have the transducer perpendicular to the skin surface (neither rocked nor tilted) such that no other angles need to be replicated. The ultrasound is subsequently brought into the field with a sterile probe cover and sterile gel is wiped away right before a spinal needle is inserted with the ultrasound trajectory approximated.

Our study was approved by the University of Minnesota Institutional Review Board. We conducted a retrospective review of all patients who underwent LP by the HMPS from October 2017 through October 2021. Before the implementation of a standard curriculum, approaches to LP varied in the use of US and patient positioning (seated vs. side‐lying). The initial US technique using the USPM approach (Figure 1) was utilized by a single hospitalist in 2018 after intensive self‐study and training with anesthesiology. After developing and standardizing a curriculum (consisting of a slide presentation and supporting documents on a cloud‐based server), each HMPS member was given access to the curriculum for self‐study and received at‐the‐elbow instruction on the USPM approach until competency was reached. This was demonstrated by at least 2–3 independently successful LPs using an USPM approach. Training proceduralists in this way occurred over the course of days to weeks depending on LP volume and proceduralist service time, with the average hands‐on time required of the trainer to be approximately 4–5 h. Over the study time period, 20 individual proceduralists on the HMPS service performed LPs. Each HMPS proceduralist underwent training starting June 2019 and all HMPS proceduralists completed training by January 2020. Subsequently, all HMPS proceduralists were encouraged to attempt all subsequent LPs using an USPM approach, with the lateral decubitus position being the recommended as the initial patient position for the USPM approach and with the understanding that clinical conditions at the bedside may demand that an alternate approach be used.

### Patients

We evaluated charts of all patients who underwent LP from October 2017 through October 2021. Charts were identified using a HIPAA secure database (Qualtrics) maintained by the HMPS, which includes all procedures, successful and unsuccessful, entered by the performing hospitalist. This data set has been maintained to track procedures and complications as well as for quality improvement and service administration purposes. LPs that were performed during the training period from June 2019 through January 2020 were excluded from analysis. In the pre‐standardization group, charts were reviewed for outcomes below and to substantiate that the use of US for a ML approach was not indicated in the documentation (i.e., documented as performed with palpation and not US).

### Outcome measurements

The primary outcome of this study was the proportion of successful attempts at LP before and after USPM technique standardization. Successful LPs were those where adequate CSF obtained to meet the patients clinical needs, as indicated by the HMPS proceduralist in their procedure documentation. Secondary analysis included the number of LP attempts, patient BMI, positioning and LP indication, referral to neuroradiology for LP completion, and complication rates. All data were manually abstracted from the Electronic Health Record (EHR) (Epic®) by chart reviewers following a standardized protocol using REDCap (Research Electronic Data Capture) electronic data capture tool.^7^, ^8^ Basic demographic data (age and sex), the primary outcome (LP success), as well as key variables of interest (BMI, indication, platelet count, INR, CSF RBC count, number of attempts, attempted location, neuroradiology referral, preprocedure history of spinal surgery, postprocedure epidural hematoma, and post procedure need for blood patch) were collected. Neuroradiology referral was recorded for cases where a consult or LP procedure note was documented after a failed bedside attempt. Charts requiring adjudication were identified by chart reviewers and adjudication was completed by consensus between the chart reviewer and the first author. In addition, 20% were also reviewed by the first author to ensure accurate data abstraction.

### Analysis

Our primary analysis compared the probability of a successful LP after USPM technique standardization versus ML approach. Our causal estimand is the difference in such probabilities averaged over our sample (i.e., the percentage point risk difference between approaches in the likelihood of a successful procedure). We estimated this risk difference by first fitting a logistic regression model that expressed the log‐odds of success as a linear function of: before and after LP standardization, patient sex, patient age, whether or not the LP was given for delivery of chemotherapy, and patient BMI. These baseline characteristics were incorporated to improve the precision of our risk difference estimate to account for other extraneous variables.

We then applied this logistic regression model to compute a predicted probability of success for each of the USPM and ML approaches for every procedure in our sample, plugging in patient‐specific values for each characteristic in the model. The difference in each predicted probability estimates an individual treatment effect. We averaged these individual treatment effects across the entire sample to estimate the average treatment effect of the USPM versus ML approaches.^9^ The standard error of this estimated risk difference used for confidence intervals and *p*‐values was computed by applying the delta method to robust estimates of the variance of the logistic regression coefficients. We used the “marginaleffects” package in R to implement this estimation and inference procedure.

Comparisons involving discrete variables, for example, the relationship between patient sex and the likelihood of a successful procedure, quantified differences in the outcome variable by levels of the predictor variable using the score test for the difference in two raw proportions. Similarly, mean comparisons of continuous variables between discrete categories used the unadjusted, two‐sample *t*‐test. The *p*‐values for secondary outcomes were not adjusted for multiplicity.

## RESULTS

The HMPS recorded 84 ML attempts over the 2‐year period before USPM LP training and 185 USPM attempts in the period following training (Table 1) with success rates of 72.6% and 85.4%, respectively (Table 2). The probability of a successful procedure was 13.6 (95% CI: 2.8, 24.4, *p* = .014) percentage points higher when compared to the pre‐USPM (or ML approach) accounting for sex, age, BMI, and if the LP was for chemotherapy (Table 3). The odds of success for USPM versus ML was also significantly higher (OR 2.4, 95% CI: 1.2, 4.8; *p* = .012) adjusting for the same covariates (Table 3). Both groups had similar periprocedural coagulation profiles, and similarly low rates of complications, such as traumatic tap, postprocedural blood patch, and epidural hematoma (Table 2). Provider experience was similar, with an average of 78.7% and 78.0% of proceduralists having at least 1 year of HMPS experience, in the pretraining and posttraining groups, respectively (Supporting Information S1: Appendix 1). For the 52 charts assessed by multiple reviewers, interobserver agreement by Fleiss' kappa values were 0.95, 1.0, 0.90, and 0.90 for LP success, reason for LP, patient position, and Neuroradiology consult, respectively. Intraclass correlation coefficients were 0.95, 0.99, and 0.90 for BMI, platelet, and INR, respectively.

**Table 1:** Pre‐lumbar puncture baseline factors.

Characteristic	Pre‐USPM training,^a^ *N* = 84	Post‐USPM training,^a^ *N* = 185
Mean age (SD), y	56.7 (14.6)	52.4 (17.5)
Female, No. (%)	28 (33.3%)	80 (43.2)
BMI, mean (SD)	25.4 (5.0)	27.5 (6.0)
Obesity (BMI ≥ 30), No. (%)	17 (20.2)	60 (32.4)
Prior back surgery, No. (%)	4 (4.8%)	4 (2.2%)
Reason for LP, No. (%)		
Intrathecal chemotherapy	12 (14.3%)	72 (38.9%)
Diagnostic	72 (85.7%)	113 (61.1%)
INR > 1.5,^b^ No. (%)	7 (9%)	11 (6.5%)
Platelets <50 K, No. (%)	7 (8.3%)	16 (8.6%)
Lateral decubitus positioning,^c^ No. (%)	41 (48.2%)	160 (86.5%)
Long spinal needle (≥ 5 inch), No. (%)	4 (4.8%)	27 (14.6%)

Abbreviations: BMI, body mass index (kg/m^2^); HMPS, hospital medicine procedure service; INR, international normalized ratio; LP, lumbar puncture; SD, standard deviation; USPM, ultrasound‐assisted paramedian.

^a^ Before USPM training all LPs were performed by a ML approach, and the majority were without any ultrasound assistance. After USPM training, a paramedian approach with ultrasound assistance was treated as the default approach for HMPS LP attempts, although proceduralists could choose to deviate.

^b^ Note that 6 (7.1%) cases in the Pre‐USPM Training group and 17 (9.2%) of cases in the post‐USPM training were missing INR data. Additionally, 9 (11%) and 10 (5.4%) were missing patient positioning data and 24 (29%) and 33 (18%) were missing spinal needle data in the pre‐USPM and post‐USPM training groups, respectively.

^c^ All LPs performed in either seated/upright or lateral decubitus positioning (for ~10% of LPs in each group positioning was not recorded).

**Table 2:** Lumbar puncture related outcomes and complications.

Outcome	Pre‐USPM training,^a^ *N* = 84	Post‐USPM training,^a^ *N* = 185
Successful procedure, No. (%)	61 (72.6%)	158 (85.4%)
CSF RBC count ≥ 10,^b^ No./Total (%)	9/59 (15.3%)	25/153 (16.3%)
Neuroradiology consulted for LP, No. (%)	21 (25.0%)	23 (12.4%)
Blood patch performed, No. (%)	1 (1.2%)	2 (1.1%)
Spinal/epidural hematoma, No. (%)	0 (0%)	0 (0%)

Abbreviations: CSF, cerebrospinal fluid; HMPS, hospital medicine procedure service; LP, lumbar puncture; RBC, red blood cell; USPM, ultrasound‐assisted paramedian.

^a^ Before USPM training all LPs were performed by a midline approach, and the majority without any ultrasound assistance. After USPM training, a paramedian approach with ultrasound‐assistance was treated as the default approach for HPMS LP attempts, although proceduralists could choose to deviate.

^b^ RBC counts were recorded from either CSF tube 3 or 4 (toward the end of CSF collection). Total indicates the total CSF samples where cell counts were analyzed.

**Table 3:** Ultrasound‐assisted paramedian LP technique effect size estimates (pre vs. post).

Assessment	Pre‐USPM training,^a^ *N* = 84*	Post‐USPM training^a^ *N* = 185*	Difference in Prob. of Success^b^ (95% CI)	Odds ratio of success^c^ (95% CI)
Primary outcome				
Success, No. (%)	61 (72.6%)	158 (85.4%)	+13.6 (+2.8, +24.4)	2.4 (1.2, 4.8)
Subgroup analysis				
Success for BMI < 30, No./Total (%)	51/67 (76.1%)	108/125 (86.4%)	+10.3 (−0.9, +22.9)	2.0 (0.9, 4.3)
Success for BMI ≥ 30, No./Total (%)	10/17 (58.8%)	50/60 (83.3%)	+24.5 (+1.8, +49.2)	3.5 (1.1, 11.5)
Secondary outcome				
Neuro IR consult for repeat LP, No. (%)	21 (25.0%)	23 (12.4%)	−12.6 (−23.7, −2.8)	0.4 (0.2, 0.8)

Abbreviations: BMI, body mass index (kg/m^2^); Neuro IR, Neuroradiology; LP, lumbar puncture; USPM, ultrasound‐assisted paramedian.

^a^ Before USPM training all LPs were performed by a midline approach, and the majority were without any ultrasound assistance. After USPM training, a paramedian approach with ultrasound‐assistance was treated as the default approach for HPMS LP attempts, although proceduralists could choose to deviate.

^b^ Difference in probability of success for the primary outcome calculated using the logistic regression model as described in the methods. Also described as the percentage point risk difference.

^c^ Adjusted odds ratio presented based on logistic regression model for the primary outcome, whereas secondary analyses are unadjusted comparisons of the raw proportions.

The post‐USPM LP group had a larger portion of patients for whom an LP was performed for intrathecal chemotherapy, which likely reflects a temporal trend toward the HMPS performing procedures instead of the Oncology service (Table 1). The probability of success in patients with BMI < 30 was 10.3% higher (95% CI –0.9, 22.9) in the post‐USPM group and for patients with BMI ≥ 30 was 24.5% higher (95% CI 1.8, 49.2) in the post‐USPM group (Table 3). The post‐USPM group also had a larger portion of patients with obesity (BMI ≥ 30) and who required longer spinal needles for CSF access.

Standardization of the USPM technique was also associated with a lower probability of neuroradiology consultation for repeat, radiography‐guided LP attempts (12.4% of LP cases in the post‐USPM LP group compared to 25.0% in the ML approach group).

## DISCUSSION

Standardization of an USPM approach was associated with a clinically meaningful and statistically significant improvement in HMPS LP success rate among hospitalized patients. To date there is no evidence evaluating the effectiveness of the paramedian approach to bedside LP using intra‐procedural ultrasound assistance in the acute care setting. However, our improvement in success rate is similar to a RCT evaluating an ultrasound‐guided paramedian approach to spinal anesthesia for elective joint replacements, where a 16% improvement in 2nd attempt success rate was observed in the paramedian approach in comparison with a palpation‐guided approach.^2^ The USPM success rate was higher than the 73%–81% success rates for US‐assisted ML approaches that have been recently reported by other HMPS groups.^9^, ^10^ We also observed a reduction in neuroradiology referral. This finding is clinically important for both patients and hospital systems, as patients are able to undergo one procedure instead of two and the hospital system will have greater availability for subspecialists to perform higher complexity procedures. The broad availability of a HMPS also likely results in a corresponding decrease in time to procedure and reduction of diagnostic delay.

While ultrasound‐assisted midline approaches to LP have repeatedly been associated with improved success rates, this has most consistently been demonstrated in high BMI populations.^11^ Evaluations of the impact in normal BMI patients with palpable landmarks have seen mixed results.^5^ In this study, we found that an effect likely persists in obese (BMI ≥ 30) and nonobese (BMI < 30) patients. Although it should be noted, in the lower BMI group the increased success rate trend did not meet statistical significance, and the study was not specifically powered to evaluate this subgroup and thus future research is warranted to validate the findings.

Multiple factors likely contribute to the associated improved success rates observed with an ultrasound‐assisted paramedian approach. First, the USPM approach utilizes the interlaminar space, which is normally wider than the interspinous space utilized by the ML approach. Second, the visualization of the target spinal level with US allows for the development of a shared mental model among trainers and trainees alike, in contrast to palpation of landmarks, which may be more operator dependent. Third, common comorbidities such as degenerative spondylolisthesis, lumbar lordosis, and encephalopathy can impair lumbar flexion and narrow the interspinous aperture; the interlaminar space is less frequently affected by these comorbidities. The paramedian ultrasound view allows the provider to identify the ideal angle of entry, estimate the needle length required to access CSF (i.e., the distance between the skin and the posterior complex or anterior complex as seen on US), correctly identify the sacrum and spinal levels, and select the most favorable interlaminar space.

Ultrasound availability and use among hospitalists is now common and as ultrasound‐assisted techniques have repeatedly demonstrated improvements in safety and efficacy, its incorporation into bedside procedures has also increased. While spinal sonoanatomy may be less familiar to most hospital medicine providers and the initial learning curve may appear steep, clinicians with a basic understanding of point‐of‐care ultrasound (POCUS) properties and US techniques will likely be able to quickly identify the key structures and correct angles needed for the USPM approach to LP with similar learning curves as other ultrasound‐assisted procedures. Despite the observed staff turnover throughout the study period, our transition to an US‐assisted technique was still associated with a significant improvement in LP success, demonstrating that our USPM technique was transferable during the HMPS onboarding process. Our HMPS mirrors that of other procedure services with varying ranges of experience and levels of previous training. Our improved success rate may actually underestimate the effect size associated with the USPM technique itself due to provider turnover and the subsequent onboarding of new proceduralists.

Our study has limitations. The study is observational, retrospective, and relied on HMPS staff to accurately record each LP in our database while actively on service. The accuracy of the periprocedural data was confirmed via a standardized chart review process, but it is possible we performed or attempted LPs during the study period that are missing as a result of providers not accurately logging each procedure. While the USPM technique was standardized and, after the training period, providers reported using this as the primary LP technique, it is possible that in some instances there was deviation to a ML approach that was not accurately reflected in the procedure notes that were reviewed. It is possible that some providers in the pre‐standardization group used a US‐assisted ML approach and did not document this in the chart. However, these instances, if present, were likely infrequent and if incidentally included would be more likely to decrease the USPM effect size. Nonetheless, caution is warranted when interpreting a retrospective study such as ours. While the HMPS maintained a similar percentage of proceduralists with at least 1 year of service experience throughout the study time period, it is possible that increasing LP case volume during the second half of the study impacted skill differential between the study groups. Our study is unable to compare US‐assisted ML and USPM success rates. Additionally, in many instances, the number of attempts were not reflected in the procedure notes and, as such, any conclusion on the technique's impact on the number of attempts is limited. Similarly, the analyses of secondary outcomes are descriptive in nature and should not be interpreted causally. Finally, patient positioning was not included in the regression model because of likely collinearity with the USPM approach, as lateral decubitus positioning was the recommended initial positioning for this technique. Lateral decubitus positioning can increase the needle depth needed, reduce the interspinous opening, and make identification of the midline more challenging, which could result in underestimation of the USPM effect size.^12^ However, an emergency medicine study that evaluated success rates between seated and lateral decubitus positions did not observe a significant difference.^13^

Future investigations should evaluate the efficacy of this technique using a prospective randomized design, comparing paramedian and midline ultrasound‐assisted techniques, and assess success rate, total attempts, and needle redirections or “passes,” as these can correlate to postprocedure back pain. Understanding which users (e.g., trainees, LP performing providers in other specialties) can adopt this technique, the effect size for those groups, and the ideal training protocol also warrants additional evaluation.

## CONCLUSION

Ultrasound‐assisted paramedian LP was associated with an increased bedside LP success rate, a reduction in neuroradiology utilization, and is a transferable skill within an active HMPS. Proceduralist groups that perform bedside LPs on hospitalized patients and who have POCUS proficiency should consider incorporating a USPM approach to their practice.

## CONFLICT OF INTEREST STATEMENT

The authors declare no conflict of interest.

## ETHICS STATEMENT

The University of Minnesota Institutional Review Board reviewed and approved this study (MOD00037126) in accordance with the DATA/Specimen Only Protocol at the University of Minnesota.

## Supporting information

Supplementary Information
