# Supplementary material for: Increased lumbar puncture success using a paramedian approach: A retrospective cohort study
Source: J Hosp Med. 2025 Jul 21;21(1):42–8. doi: 10.1002/jhm.70138 (PMC12747465; doi:10.1002/jhm.70138)
Supplement: Supplementary file 1 — Supplementary Information [file JHM-21-42-s001.docx]

Appendix 1. Experienced vs Non Experienced Providers

| **PRE STANDARDIZATION** | **Experienced Proceduralists** | **Total** | **Percentage Experienced Proceduralists** | **Experienced Providers** | **Providers <1 year** |
| --- | --- | --- | --- | --- | --- |
| 2017-2018 | 8 | 11 | 72.7% | Lambda, Omicron, Chi, Pi, Alpha, Tau, Zeta, Nu | Epsilon, Theta, Phi |
| 2018-2019 | 11 | 13 | 84.6% | Lambda, Omicron, Chi, Pi, Epsilon, Phi, Tau, Zeta, Nu  Beta, | Psi, Sigma |
|  |  |  | **78.7%** |  |  |
| **POST STANDARDIZATION** |  |  |  |  |  |
| 2019-2020 | 10 | 14 | 71.4% | Lambda, Omicron, Epsilon, Phi, Theta, Zeta, Nu  Beta,  Psi, Sigma | Mu, Gamma, Delta, Xi |
| 2019-2020 | 11 | 13 | 84.6% | Lambda, Omicron, Epsilon, Phi, Theta, Zeta,  Psi,  Mu, Gamma, Delta, Xi | Upsilon, Rho |
|  |  |  | **78.0%** |  |  |
